# Regulation of CD4^+^ and CD8^+^ T Cell Biology by Short-Chain Fatty Acids and Its Relevance for Autoimmune Pathology

**DOI:** 10.3390/ijms23158272

**Published:** 2022-07-27

**Authors:** Carmen Schiweck, Sharmili Edwin Thanarajah, Mareike Aichholzer, Silke Matura, Andreas Reif, Elske Vrieze, Andreas Weigert, Alexander Visekruna

**Affiliations:** 1 Department for Psychiatry, Psychosomatic Medicine and Psychotherapy, University Hospital Frankfurt-Goethe University, 60528 Frankfurt, Germany; sharmili.edwinthanrajah@kgu.de (S.E.T.); mareike.aichholzer@kgu.de (M.A.); silke.matura@kgu.de (S.M.); andreas.reif@kgu.de (A.R.); 2 Department of Psychiatry and Neurosciences, UPC KU Leuven, Campus Gasthuisberg, KU Leuven, 3000 Leuven, Belgium; elske.vrieze@uzleuven.be; 3 Institute of Biochemistry I, Faculty of Medicine, Goethe-University Frankfurt/Main, 60590 Frankfurt, Germany; weigert@biochem.uni-frankfurt.de; 4 Institute for Medical Microbiology and Hygiene, Philipps-University Marburg, 35043 Marburg, Germany; visekrun@staff.uni-marburg.de

**Keywords:** short chain fatty acid, immune system, T cell, intervention, RCT

## Abstract

The gut microbiota encodes a broad range of enzymes capable of synthetizing various metabolites, some of which are still uncharacterized. One well-known class of microbiota-derived metabolites are the short-chain fatty acids (SCFAs) such as acetate, propionate, butyrate and valerate. SCFAs have long been considered a mere waste product of bacterial metabolism. Novel results have challenged this long-held dogma, revealing a central role for microbe-derived SCFAs in gut microbiota-host interaction. SCFAs are bacterial signaling molecules that act directly on host T lymphocytes by reprogramming their metabolic activity and epigenetic status. They have an essential biological role in promoting differentiation of (intestinal) regulatory T cells and in production of the anti-inflammatory cytokine interleukin-10 (IL-10). These small molecules can also reach the circulation and modulate immune cell function in remote tissues. In experimental models of autoimmune and inflammatory diseases, such as inflammatory bowel disease, multiple sclerosis or diabetes, a strong therapeutic potential of SCFAs through the modulation of effector T cell function was observed. In this review, we discuss current research activities toward understanding a relevance of microbial SCFA for treating autoimmune and inflammatory pathologies from in vitro to human studies.

## 1. Introduction

Aberrant immune responses are the core mechanism behind several autoimmune pathologies such as rheumatoid arthritis, multiple sclerosis, inflammatory bowel disease and diabetes. Regulating the delicate balance of inflammatory mediators is key for treatment of these pathologies. Next to monoclonal antibodies, non-steroidal and steroidal anti-inflammatory drugs, in recent years, the gut microbiota has received much attention as treatment target for autoimmune pathologies [1]. Increasing awareness of the bidirectional role the gut microbiota plays in the communication with both the brain and periphery has prompted several studies investigating various treatment options to modify the gut microbiome and thus tamper inflammation. These studies include dietary interventions including pre-, pro- and synbiotics, as well as fecal microbial transplants (e.g., clinical trial registration numbers: NCT03766321, NCT04038619, NCT03766321, NCT04574024). One converging agent that may be behind the mechanism for this modulation by the above-mentioned treatments, are short-chain fatty acids (SCFAs). SCFAs are implicated in immune cell metabolism and regulation and can have both beneficial and deleterious consequences for inflammation, depending on the type, site of action, milieu, dose, target cell type and duration of exposure. While it is understood that SCFAs modulate the immune system through their action on epithelial cells [2,3] the past decade has also revealed a stimulating effect on B cells [4], Immunoglobulin A (IgA) responses [5] and diverse T cell subsets [6,7]. Modulation of pathogenic T cell activity is a major focus of interest for treatment of autoimmune disorders and several intervention studies have been published in the past 3 years. To our knowledge, no review summarizes the current literature on the effect of SCFA interventions on CD4^+^ and CD8^+^ T cell responses in animal and human studies of the above-mentioned autoimmune disorders. Therefore, we here examine the recent literature on butyrate, acetate, propionate and valerate and their impact on T helper and cytotoxic T cell biology.

### What Are Short-Chain Fatty Acids?

Saturated fatty acids with a maximum of 5 carbon atoms are called SCFAs and are defined by the number of carbon atoms present. Among the SCFAs, formate (C1), acetate (C2), propionate (C3), butyrate (C4), and valerate (C5) take the center stage in the scientific literature. In humans, acetate, butyrate and propionate are found in higher concentrations than valerate or formate [8]. SCFAs are produced through fermentation of undigested carbohydrates by anaerobic bacteria in the colon [9]. In the case of acetate, it can also be derived from hydrogen and carbon dioxide or formic acid by acetogenic bacteria [10]. Since microbes provide the essential enzymes for SCFA production [11], a healthy gut microbiome is essential for immune homeostasis. SCFAs are used as energy source for colonocytes [12] and the microbiota [13], but can also have potent immunological properties. For instance, SCFAs can influence (i.e., both induce and reduce) the production of cytokines by epithelial cells [14,15]. Recently, it was reported that SCFAs can also influence T cell responses [7].

Most SCFAs are absorbed in the gut and only a small portion (estimated at around 5%) is excreted [16]. SCFAs thus may likely have the strongest influence on (T) cells locally in the colon, where concentrations can reach up to ~130 mmol/kg in the caecum [8]. However, SCFAs also enter the circulation via the blood stream in lower, though still detectable concentrations. Particularly acetate, and to a lesser extent propionate and butyrate, are found in the blood stream of healthy people (from around 20–500 μmol/L for acetate, 1–13 μmol/L for propionate and 1–12 μmol/L for butyrate) [8,9,17,18] and may thus also exert systemic effects (see Table 1). Given that the site of action (e.g., local versus systemic) differs considerably in terms of exposure, cell types involved and the milieu, in the following we point out where and under which circumstances the different effects occur.

## 2. Effect of SCFAs on General Function of T Cells in the Gut and Periphery

Two major subclasses of T cells are T helper and cytotoxic T cells. T helper cells are defined by the expression of the cluster of differentiation (CD) 4 that, together with the specific T cell receptor, allows the recognition of peptides presented on the Major histocompatibility complex (MHC) class II e.g., [20]. Cytotoxic T cells express CD8 next to the T cell receptor, recognize peptides on MHC class I and are particularly important for intracellular pathogen recognition. CD4^+^ T cells are involved in shaping adaptive immune responses and have highly specialized functions with a primary function in extracellular antigen recognition. Upon activation, Th cells differentiate into discrete, but partly interconvertible subsets with specific functions, such as Th1, Th2, Th17 and regulatory T cells (Treg). Two types of T helper cells play a particular role in the delicate balance of homeostasis and prevention of inflammation, particularly in the gut: T regulatory and interleukin (IL)-17 producing T cells. Given the scope of this review, we here focus majorly on CD8^+^ cells, Tregs and two subsets of IL-17 producing cells: T helper (Th) 17 and γδ T cells.

### 2.1. Effect on Tregs

Current evidence indicates that SCFAs can have an anti-inflammatory effect which can be exerted via different mechanisms (for a review see [2]). Next to strong effects on epithelial and dendritic cells, macrophages and neutrophils, SCFAs have a strong IgA inducing effect [4,5] and promote Treg differentiation and responses [6,7,21,22].

Forkhead box P3 (Foxp3) positive regulatory T cells recognizing commensal epitopes (cTregs) maintain homeostasis by promoting tolerance through IL-10 production, prevention of intestinal inflammation [23] and tissue repair [24]. In contrast, Th17 cells can mediate both regulatory and inflammatory functions [25] and can secrete high amounts of IL-17. Th17 cells are considered to have a classical T cell receptor, as most T cells, which is composed of an α and β chain, whereas the T cell receptor of γδ T cells is composed of a γ and δ chain. γδ T cells are unconventional T cells, since unlike the classically adaptive αβ T cells, they unite adaptive and innate immune characteristics [26]. Their innate function is exemplified by their ability to be activated independent of their T cell receptor recognizing the MHC–antigen complex. Instead, they respond very rapidly to innate stimuli such as cytokines, secreting in turn various cytokines themselves, including IL-17. The large majority of γδ T cells is tissue resident [27]. This T cell type is particularly high in the gut [28] and may thus play an important role regarding the immune balance where IL-17 is involved.

**Figure 1:**
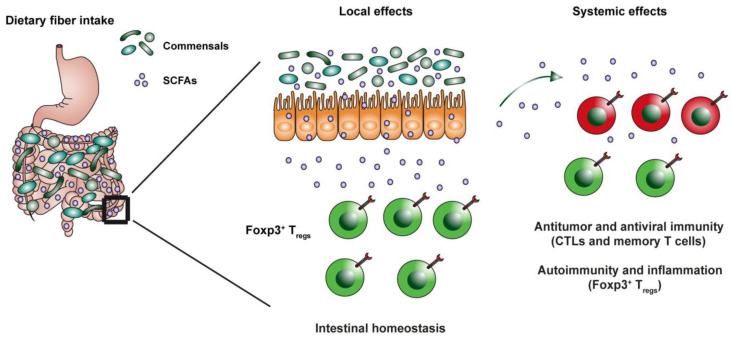
Bacterial fermentation of dietary fiber in the intestinal lumen leads to generation of SCFAs such as acetate, propionate, butyrate and pentanoate. Microbial SCFAs are potent signaling molecules that are capable of crossing the epithelial barrier and of acting directly on immune cells in the lamina propria. SCFAs promote the epithelial barrier function, local expansion of colonic Tregs and mucosal IgA responses. Microbiota-derived molecules such as SCFAs are also able to reach the circulation and to influence the host health by strengthening anti-viral and anti-cancer T cell responses as well as suppressing autoimmunity and inflammation in peripheral tissues.

A disturbed balance between regulatory and IL-17 producing cell types, which are highly concentrated in the lamina propria, is thought to play a crucial role in autoimmune pathology and intestinal inflammation [29]. It is understood that specific strains of commensal bacteria can induce Treg and/or Th17 cell expansion and differentiation (e.g., [30,31,32], but whether this is due to a direct response to specific bacteria or rather to their by-products was incompletely understood until the early 2000s. In 2013, Furusawa showed that differentiation of Tregs can be directly induced by the SCFA butyrate, both in vivo and in vitro, even under Th1 or Th17 polarizing conditions. The luminal concentration of butyrate also correlated with the number of Tregs [22]. Adding to this, Arpaia et al. (2013) further found that this increased differentiation of Tregs after butyrate exposure was due to peripheral induction, and not increased thymic output [21]. Interestingly, the increased differentiation of Tregs could be observed for butyrate and in the periphery also for propionate, but not for acetate [21,22]. Around the same time, Smith et al. showed that SCFAs which were orally administered to germ-free mice for 3 weeks, increased the number of cTregs. However, this effect was not observed in Treg populations found in the mesenteric lymph nodes, spleen or thymus [6]. These findings show that the specific local effects of SCFAs on Treg differentiation and colonization do not necessarily extend to the periphery, be that due to exposure to different doses, higher expression and thus regulation of GPR43 in colonic Tregs [6] or other mechanisms (see Figure 1).

### 2.2. Effect on Effector Th17 Cells

Often considered as counterbalance of T regulatory cells, IL-17-producing Th17 cells can induce tissue inflammation in auto-immune responses, but they are also crucial for combating pathogen infection and exert protective effects [33,34]. These functions partly depend on the different cytokines produced by Th17 cells: IL-21 promotes IgA production, while IL-22 is thought to induce tissue repair and increase the epithelial barrier function [34]. Regarding the modulation by SCFAs, Park and colleagues (2015) observed an acetate- and propionate- induced, dose-dependent increase in Th17 and Th1 cells depending on the cytokine milieu. This was also observed in the spleen and mesenteric lymph nodes [7]. This finding is important since it suggests that the observed effect is not locally restrained. Increased Th1 and Th17 cells are often linked to increased auto-immune pathology and would thus be detrimental for disease progression. However, when acetate-treated cells were transferred to induce colitis in mice, the Th17 cells only induced mild inflammation and were not as pathogenic as control effector T cells [7]. This was also observed by others and is likely linked to the simultaneous high production of IL-10 by these Th17 cells [35]. Most importantly, the clinical relevance of this finding becomes clearer when considering human studies. Production of IL-17 by human Th17 cell derived from patients with multiple sclerosis, or by γδ T cells from patients with inflammatory bowel disease (IBD) can be strongly suppressed by supplementation of SCFAs such as propionate (see below for a more detailed overview) [36,37].

### 2.3. Effect on CD8^+^ Cells

SCFAs can also exert a potent antiviral function and can regulate autoimmunity and inflammation, partly via the regulation of cytotoxic CD8^+^ cell subsets (see Figure 1). For instance, Trompette et al. (2018) showed that oral administration of SCFAs conferred protection to influenza infection (i.e., improved clinical scores and enhanced survival rates) by reducing neutrophil infiltration, reducing tissue damage and increasing CD8^+^ T cell activation [38]. Interestingly, Bachem et al., also found that butyrate potentiates metabolism and improves anti-viral immunity of CD8^+^ T cells in the spleen and liver. This process was suggested to happen by boosting CD8^+^ memory T cell survival and activation through increased glycolysis and increased acetylation of glyceraldehyde-3-phosphate dehydrogenase [39]. Next to their anti-viral properties SCFAs can also exert anti-tumor effects [40,41]. Since SCFAs serve as important metabolic energy source for cells, and given the fact that in the tumor microenvironment T cells compete for glucose with cancer cells, Qiu and colleagues explored whether in vitro, acetate could restore CD8^+^ responses [42]. The authors showed that acetate was able to rescue hypo responsive CD8^+^ T cell functions by increasing cytokine production and histone acetylation but did not affect lactate production or cell proliferation.

The reinforcing effect of SCFAs on CD8^+^ T cell responses is not limited to acetate. He et al. (2021) have shown that butyrate increases CD8^+^ T cell antitumor responses in the tumor microenvironment [43]. Luu and colleagues demonstrated that both butyrate and pentanoate improved immunotherapy for cancer by boosting CD8^+^ T cell and chimeric antigen receptor (CAR) T cell responses [44]. Interestingly, butyrate concentrations also correlated with clinical outcomes. Responders to oxaliplatin, which is used for treatment of colorectal cancer, had higher levels of butyrate than non-responders [43]. It is now clear that the microbiome is crucially involved in the efficacy of anti-tumor immune therapies [45], a process in which SCFAs may play a decisive role. However, randomized clinical trials are needed to make definite statements as to the efficacy of SCFAs alone or in combination with established therapies in patients with cancer.

### 2.4. Anti-Inflammatory Effect in Periphery Is Dose-Dependent

As shown in the Figure 1, current evidence suggests that SCFAs generally induce an anti-inflammatory effect in the gut, and potentially in the periphery. Several of the above-mentioned studies found different effects based on the dose of SCFAs.

Delivering SCFAs in mouse models is usually performed by administration in the drinking water, oral gavage, or intraperitoneal injection. Since SCFAs can be derived from naturally occurring fibre, dietary interventions including fibre, pre- and probiotics are a convenient means to increase peripheral SCFA concentrations in humans. Other options to deliver SCFAs include enemas, capsules or even delivering microbial strains that are thought to produce SCFAs. Blaak et al. (2020) recently reviewed the literature regarding in vivo studies assessing the concentrations that can be found in the colon and periphery and as others (e.g., Cummings et al., 1987), have reported much lower concentrations in the periphery than in the colon [8,9].

The concentration of most SCFAs in the peripheral bloodstream is approximately between 1–15 μmol/L [8,9,18] at the exception of acetate, which can reach concentrations up to 500 μmol/L [17] (see Table 1). However, concentrations in the colon and those used for in vitro studies are often around 100 µM or higher (e.g., [6,21]. Importantly, the effect of SCFAs on Th1/Th17 cells described by Park and colleagues occurred only at relatively high concentrations (e.g., 5 mM for acetate and 0.5 for propionate, see below), but not at levels below this threshold [7]. It therefore stands to question whether the same effects seen in vitro can be induced in vivo in animals or humans, using interventional strategies. Qi Hui et al. (2021), investigated the effect of 2 μmol, 20 μmol and 200 μmol butyrate on cytokine production in human peripheral blood mononuclear cells (PBMCs) stimulated with lipopolysaccharide [46]. Levels of IL-6 and IL-1ß were significantly reduced at concentrations of 20 μmol, whereas concentrations of 2 μmol did not show any effect. Similar effects were observed for propionate, for IL-1ß and TNF-α, but no effect was observed for acetate. Coutzac et al. (2020) reported that the increase of Tregs after CD3 stimulation in vitro was found with butyrate concentrations of 50 µM or 100 µM, but not at concentrations of 10 µM [47]. This may suggest that physiological concentrations can potentially be sufficient to induce an anti-inflammatory effect, but especially for butyrate and propionate the concentrations would be below the window of efficacy. It should however also be noted that most studies reported no effect for acetate when butyrate or propionate were effective. See Table 2 for an overview of concentrations used in different studies.

A second question is what concentrations of SCFAs can be reached in the peripheral blood after oral ingestion, which is arguably the preferred intervention method in humans. A study in healthy controls ingesting daily concentrations of oral capsules with high-dose, colon-delivered acetate (174.2 mmol), propionate (13.3 mmol) and butyrate (52.4 mmol) found that serum concentrations of acetate, butyrate and propionate nearly doubled after a 1-week intervention, reaching concentrations of 84.08 μM, 1.40 μM and 2.04 μM respectively [52]. Although increased, the level in sera after such an intervention may still be too low to reach efficacy in peripheral blood. This does naturally not mean that SCFAs cannot have a significant impact in the colon, however in humans, as biopsies are rare, this is more difficult to assess.

## 3. SCFA Interventions in Inflammatory Disorders

Given the potent immune-modulatory functions demonstrated above, the question arises whether SCFAs may be instrumental in modulating unwanted or exaggerated immune responses, as seen in autoimmune pathologies. In the following we will examine animal and human studies which assessed CD4^+^ and CD8^+^ T cell function upon SCFA exposure with an emphasis on autoimmune pathologies.

### 3.1. Multiple Sclerosis

Several studies have investigated the association of serum SCFA concentrations, inflammatory proteins and circulating T cell subsets in humans with and without disease. Some evidence for the beneficial effect of SCFAs supplementation exists for multiple sclerosis (MS). MS is a central nervous system (CNS) autoimmune disease with a role for autoreactive B and T cells: T cell subsets implicated in MS are particularly cytotoxic CD8^+^ cells, Th1 and Th17 cells [53]. The association between levels of SCFAs and MS has been shown in several association studies. Low levels of serum/plasma propionate, acetate and butyrate were found in patients with clinically isolated syndrome or MS compared to controls [54,55]. However, others reported contradictory findings or no changes for acetate, propionate, butyrate or valerate in blood or stool samples [17,56]. Only few studies have investigated the number of circulating cells in relation to SCFAs. In a small sample no association was found between levels of acetate, butyrate or propionate with circulating Treg, Th1 or Th17 cells, but a small positive association of T follicular cells with serum butyrate and propionate levels was detected [55]. Pérez-Pérez and colleagues showed higher levels of acetate in blood of MS patients, which correlated with IL-17 producing CD8^+^ T cells [57]. Lastly, Zeng et al. (2019) detected a positive association between fecal concentrations of SCFAs and numbers of peripheral Tregs [58]. For an overview of association studies see Table 3.

Next to these association studies, attempts have been made to address the modulating effect of SCFAs on T cell subsets, both in autoimmune models in animals and autoimmune pathology in humans. Fatty acids have long been known to modulate disease activity in MS: While long chain fatty acids are thought to exacerbate autoimmune activity by favoring differentiation of Th1 and Th17 cells [50], SCFAs may have a beneficial effect. Haghikia and colleagues (2015) found that propionate not only increased the frequency of Tregs derived from naïve T cells, but also changed the proliferation of previously differentiated Tregs and increased IL-10 (but not IL-17) production [50]. Haase et al. (2021) recently showed that this beneficial effect of propionate may be particularly useful in case of a high-fat diet. In their experimental autoimmune encephalitis (EAE) mouse model, high fat diet aggravated the disease progression, but propionate prevented this effect [66]. The authors found that in this context, propionate particularly reduced Th17 cell-related inflammation and increased Treg function and numbers [66]. Importantly, the authors were able to show that in a small trial, a 90-day treatment of propionate also increased Treg counts (in non-obese MS patients) and reduced Th17 cell count (in the obese patient population). One of the few other studies in humans with MS, which focuses on a specific SCFA, showed that in patients with MS, short-term oral administration of propionate for 14 days led to a significant reduction in Th17 cells and an increase in Treg counts. Long term treatment (e.g., follow-up between 1-3 years) improved clinical outcome as measured by disability status and relapse rates [37]. Importantly, Haghikia et al. (2015) also demonstrated that the timing of SCFA administration is crucial, since SCFAs had a preventive effect if given at the time point of immunization in their EAE mouse model but had no effect at the time of disease onset, suggesting that preventive administration may be the key and indicating that SCFAs may generate tolerance towards the model antigen [50]. However, these results await confirmation human studies with larger patient collectives. In a nutshell, the first evidence summarized here suggests that propionate may have the potential to reduce Th17 numbers and increase Treg frequency and functionality. If this is due to regulating IL-10 secretion or other mechanisms of immune tolerance remains to be determined. Further controlled intervention studies in humans are needed.

### 3.2. Type I Diabetes

One of the autoimmune disorders with the strongest evidence for a pathogenic role of T cells is Type 1 diabetes (T1D). In contrast to Type 2 diabetes, where peripheral cells become resistant to insulin over time [67], in T1D, pancreatic insulin-producing beta cells are destroyed by autoreactive T cells early on, with a particular pathogenic role of CD4^+^ and CD8^+^ cells [68]. First evidence for decreased SCFA concentrations also exists for T1D [59,60], although further studies are needed to replicate this effect. Strong evidence for a positive effect of a SCFA intervention on T1D comes from first animal and human studies: In 2017, Mariño et al. (2017) made several ground-breaking observations: In a non-obese diabetic (NOD) mouse model of type I diabetes, a 5-week special diet designed to release high amounts of either acetate or butyrate led to higher levels of SCFAs in feces and blood [69]. Interestingly, combining both diets prevented the development of T1D in the NOD mouse, likely involving different cellular mechanisms. However, this was only true at relatively high ingested concentrations (i.e., 15%), but not at a lower dose (e.g., 7.5%) [69]. Interestingly, the acetate-rich (and, to a lower extent, the butyrate-rich) diet reduced the frequency of autoreactive CD4^+^ and CD8^+^ T cells, and led to expansion of CD4^+^Foxp3^+^ Treg cells both in the colon and spleen, but not in peripheral lymph nodes [69]. The diets were also able to indirectly reduce autoreactive T cells. Upon adoptive transfer of T cells from mice fed either with a control diet, acetate-rich diet or butyrate-rich diet, into mice with severe combined immunodeficiency, it was particularly the butyrate rich-, but not the acetate-rich diet which delayed diabetes onset [69]. The butyrate rich diet conferred its protective effect in the adoptive transfer model to the immune-deficient mice by enhanced Treg function and differentiation of Tregs from naïve T cells. The authors emphasized that since acetate seemed to primarily reduce effector T cells and butyrate increased regulatory subsets. The combination of both thus may yield the strongest effect.

Recently, Bell et al. (2022) partly confirmed these results in humans with T1D: using a 6-week intervention with a dietary combined butyrate and acetate enhancing supplement comparable to the animal study by Mariño et al. (2017). They demonstrated an increase of fecal and peripheral SCFA concentrations after treatment [70]. While the change of T and B cell subsets was not identical to the data of Mariño et al., they did observe a shift in B cells after 6 weeks of treatment and a delayed effect for B and T cells, 6 weeks after completion of the treatment (follow-up). At 6 weeks, total (naïve) B cells were increased, alongside decreased expression of the costimulatory molecule CD86. At 12 weeks, T-cell subsets expressing the inhibitory CTLA-4 expression in were increased. Together, these results could be indicative of a more tolerogenic phenotype in both cell subsets. However, the majority of the effects on T-cells were delayed and only observed at follow-up. In addition, surprisingly, a significant decrease in central memory and naïve Tregs was observed at follow-up and no significant changes in the frequency of circulating Tregs were found at 6 weeks [70]. In another clinical trial with 30 patients affected by T1D, 4g sodium butyrate or placebo were administered for 4 weeks [71]. The authors could not find any effect neither on peripheral immune cells or cytokine production, nor on CD4^+^ or CD8^+^ T cell subsets. However, they reported a reduction in autoreactive antigen-specific CD8^+^ T cells, but since this could only be observed in a small subset with large individual variability, the authors concluded that oral butyrate may not have a beneficial effect on immune cells in T1D patients and is not in line with previous animal studies [71]. Another clinical trial using a dietary supplement in a cross-over design to assess change in gut microbiome, SCFA levels and glycemic control is currently underway [72] and will shed further light on the clinical implications. To summarize, while animal studies showed promising results for SCFA in T1D these could not or only partially be replicated in humans.

### 3.3. Rheumatoid Arthritis and Short Chain Fatty Acids

Rheumatoid arthritis (RA) is an autoimmune disease which affects the joints and leads to bone loss. T and B cells are thought to infiltrate the synovium and thus affect disease progression via release of pro-inflammatory mediators. Regarding T cell subtypes, especially CD4^+^ Th1 and Th17 cells have been shown to be pathogenic [73], but there may also be a role of CD8^+^ T cells [74]. A recent study assessing levels of SCFAs in blood and stool of patients has shown reduced levels of SCFAs in patients with RA compared to controls, although diverging effects were detected for serum and stool concentrations and per study [62,63]. Others have suggested that high levels of SCFAs may provide a protective effect in individuals at risk for RA development [61,75]. Animal studies have also suggested a beneficial role of higher SCFA concentrations: Takahashi et al. (2021), using a collagen-induced arthritis (CIA) and an SKG (i.e., T-cell mediated) mouse model for RA, showed that in vitro, butyrate increased follicular T regulatory cells, which reduce secretion of cytokines necessary for B cell activation and class switching. This in turn was linked to a more favorable disease outcome [76]. In line with this, Lucas et al. (2018) investigated the effect of SCFAs in two murine models of RA (CIA and K/BxN serum-induced model) and found improved bone homeostasis (i.e., decreased osteoclast differentiation and bone resorption), and beneficial effects on disease severity after an 8-week oral supplementation of acetate, propionate or butyrate [77]. The treatment enhanced serum levels of SCFAs and, as reported by others, the numbers of regulatory T cells were increased post-treatment. Interestingly, Lucas et al. (2018) also found that serum levels of TNF-α, IFN-γ and IL-10 were increased after butyrate treatment, but not after acetate or propionate treatment, suggesting differential systemic effects [77].

Yao et al. (2022) assessed levels of SCFAs in stool samples, B regulatory cells (Bregs) and Tregs (defined as CD4^+^CD25^+^) in 9 RA patients and 10 healthy volunteers [63]. Levels of acetate, propionate, butyrate and valerate were lower in patients compared to controls, and numbers of Tregs and Bregs were also reduced. However, SCFA levels correlated to Breg numbers, but not to Tregs. The authors went on to administer a treatment of SCFAs (either butyrate, acetate or propionate alone, or a combination) in a CIA RA mouse model before disease onset. They found that all SCFAs combined had the most beneficial effect on immune cell infiltration, joint swelling and arthritis scores and increased Breg frequency [63]. Congruent with the results observed in humans, SCFA levels correlated with Breg numbers in the periphery, but not with Treg numbers. In mice, IL-6, IL-1ß, TNF-α were downregulated, and IL-10 was upregulated in treated mice. Up until now, few human intervention studies have been conducted in RA. Häger et al. (2019) performed a dietary intervention in 36 patients with RA currently receiving treatment with disease-modifying anti-rheumatic drugs (DMARDs). They administered either high-fiber bars or cereals for 28 days [65]. After treatment, the authors observed higher numbers of peripheral Tregs, and improved Th1/Th17 ratios in those with the high-fiber diet. These observations went alongside decreased IgA antibody levels, and improved quality of life. From the same study, Dürholz et al. (2020), report on a short term (30 day) intervention study with high dietary fiber intake in 29 patients with RA currently taking DMARDs and 10 healthy controls [64]. Levels of butyrate, propionate and acetate increased significantly in the serum of patients. Most cytokine levels remained unchanged, but chemokine-ligand 2, IL-18 and IL-33 decreased significantly [64]. In conclusion, first human trials have shown a beneficial effect of SCFAs in RA patients that were previously observed in animal models. Further studies are needed to corroborate these findings.

### 3.4. Inflammatory Bowel Disease

Inflammatory bowel disease (IBD) comprises diseases causing inflammation in the gastrointestinal tract- including ulcerative colitis (UC) and Crohn’s disease (CD). Since aberrant immune cell function and infiltration has been shown for IBD [78], SCFAs may provide a targeted approach to regulate immune function, particularly given to proximity to their natural source. Overall patients with CD or active UC showed lower levels of propionate, acetate, butyrate and valerate, while those with UC in remission had levels comparable to controls, or even higher levels in the case of butyrate (for reviews see [58,79]). Given this apparent deficiency, it was early on assumed that SCFAs could have a beneficial effect in IBD. However, intervention studies up to date showed controversial effects. Several early studies have reported clinical improvement in patients with distal colitis treated with a 2-week [80] or 6-week [81] topical administration of SCFAs compared to placebo. In contrast, others did not observe any difference between placebo or verum condition in RCTs after a maximum of 6 weeks treatment with butyrate [82,83]. Indeed, a recent systematic review including 8 studies concluded that treatment with butyrate enemas did not improve disease outcome in UC, while no reliable data was available for CD [84]. We would like to direct readers to this current review for an overview on SCFA interventions in IBD.

When looking at the effects on immune cells, Berndt et al. (2012) even reported a worsening of induced colitis in a dextran sulfate sodium (DSS) mouse model after oral administration of butyrate [85]. Kespohl et al. (2017) investigated this question further and showed that the environment and dosage are crucial modulators of the SCFA induced effects [51]. Under steady state conditions, lower concentrations (100 or 200 mM) of butyrate administered as a 3-week oral treatment in mice did increase differentiation of Treg cells in the lamina propria. However, Treg numbers in the spleen or mesenteric lymph nodes were unchanged, and Th1 or Th17 numbers unaffected. Interestingly, when colitis was induced, orally administered butyrate at 100mM did not increase Treg numbers, but increased expression of T-bet and IFN-γ [51]. In line with this, Trapecar et al. (2020) noted that the environment and disease pathology are essential for these effects using a multi-organ model of UC [86]. In their experiments SCFAs did reduce innate immune activation in tissues; yet, depending on the condition, also increased effector activity of CD4^+^ T cells, which in turn aggravated inflammation [86]. In a recent trial with oral SCFA administration, Lee et al. (2022) showed an increase in both Treg and Th17 cells in the colon of mice subjected to a DSS-induced model of IBD after a 14-day oral administration of butyrate or a mix of butyrate, acetate and propionate, but did not observe attenuation of inflammation [87].

Opposing to the above-mentioned findings, Park et al. (2015) noted that although numbers of effector T cells were increased after SCFA treatment, the cells were less pathogenic and induced milder forms of colitis. Importantly, in the absence of an active immune response, no change of Th1 or Th17 cells was observed after acetate administration in the drinking water, but IL-10^+^ cells were increased in the cecum. However, during an induced infection, significantly enhanced frequencies of IL-17^+^ and IFN-γ^+^ cells were observed in cecum and spleen [7]. In line with this, Sun and colleagues found SCFAs to induce IL-10 production by Th1 cells, which limited the pathological effect of induced colitis. Additionally, in vitro experiments with cells of patients with IBD showed an induction of IL-10 and decrease of IL-17 production after culture with butyrate [88]. These findings suggest that attention should be paid to the differential effects of SCFA concentration, as well as the milieu: during inflammatory conditions SCFAs may not necessarily have a dampening effect. It should also be noted that some studies have observed unwanted T cell mediated renal inflammation after oral administration of SCFAs at a concentration exceeding physiological levels, thus warranting caution with oral administration [89]. However, Marzocco et al. (2018) conducted an open label, dietary intervention study in patients with end stage renal disease receiving hemodialysis and showed improved signs of inflammation. After 12 weeks, a significant decrease in CRP, IL-2, IL-17 was observed, while IL-10 was significantly increased. No change was observed for IL-6, IFN-γ and TNF-α [90].

A recent article investigated the effect of SCFAs on γδ T cells in IBD [36]. This T cell type has been shown to be enhanced in the peripheral blood of post-infection IBD [91] and it was shown that their inflammatory activity as measured by IL-17 and IL-22 production, can be modified by the gut microbiota [36]. They also found a discrepancy in distribution of IL-17 producing γδ T cells in the small intestine or caecum/colon of mice, which they linked to the production of short SCFAs. SCFAs, in particular propionate, inhibited IL-17 production by γδ T cells, while not affecting αβ T cells (IL-17 production in the overall tissue was actually increased) ex vivo [36]. However, in vivo this effect was only found in mice with an antibiotic-induced disruption of the microbiome, but not control mice: A 3-week oral intervention with propionate did not change γδ T cells in the control population, but reduced IL-17 production by γδ T cells in the antibiotic-treated group. Interestingly, the authors also replicated this effect in human material of IBD patients [36]. Lastly, Magnusson et al. (2020) recently showed that butyrate inhibited the activation and the proliferation of both circulating and intestinal human T cells in healthy controls as assessed in biopsies [92]. However, they also showed that butyrate did not downregulate cytokine secretion in inflamed tissues of patients, indicating that context and disease-dependent effects may occur [92]. To summarize, evidence for oral SCFA interventions in IBD is controversial. Data from mouse models generally suggest a dampening effect on inflammation, depending on dose and milieu, but human data in controlled trials is still sparse. These findings highlight the importance of assessing dose- and context specific factors. Disease state, concomitant medication, inflammatory status and nutrition should be taken into account in future studies. Table 4 provides an overview of all intervention studies.

## 4. Conclusions

Some human bacterial commensal species and their metabolites have been described to promote anti-inflammatory effects and suppress the over activation of the immune system. SCFAs are the major and well-characterized class of commensal-derived metabolites, are capable of regulating metabolic and epigenetic processes in T lymphocytes and may thus maintain immune homeostasis and protect against immune-pathogenesis. In vitro studies have shown that at the right dose, SCFAs initiate differentiation of Tregs, and concurrently suppress development of pathogenic Th17 cells. SCFA-dependent regulation of Treg and Th17 cell plasticity crucially contributes to immune homeostasis in the intestine. In this review we reported on recent intervention studies in animals and humans, suggesting that SCFA-based interventions in humans and mice have the potential to shape the immunological environment in various tissues and may protect from autoimmune disorders such as multiple sclerosis, rheumatoid arthritis, and diabetes. While the first few studies in humans do report favorable outcomes for RA and MS, this effect could not be observed for interventions in T1D, and SCFA can also induce inflammatory T cell subsets, depending on the local milieu. Most current evidence in humans comes from open label studies and concentrations of SCFAs observed in the periphery are likely below the threshold to replicate the potent effects observed in in vitro studies, albeit more moderate effects have been observed. The effect of SCFA interventions on clinical symptoms and peripheral circulating immune cells is still unclear and needs further investigation in randomized controlled trials. Furthermore, besides SCFAs, several other microbiota-derived molecules such as secondary bile acids, tryptophan catabolites and polyamines are potentially able to modulate the mucosal immune system. Similar to SCFAs, tryptophan metabolites generated by commensal bacteria appear to affect intestinal T cell differentiation and impact immune responses. Thus, next to the commensal microbiota themselves, specific, diffusible molecules such as SCFAs exhibiting a potent immuno-regulatory activity should be therapeutically exploited in the future clinical trials. The next step in establishing promising biotherapeutic agents derived from the human microbiome is to decipher more thoroughly complex interactions of microbiota-derived molecules with different immune cell subtypes.

## Figures and Tables

**Table 1:** Physiological concentration of SCFAs in the healthy human colon and blood (fasted), as reported in the literature. Note that higher concentrations may be found in arterial blood compared to venous blood [19].

	SCFA	Colon (Caecum)	Blood (Serum/Plasma, Fasted)	References
*Healthy Humans*	Total SCFA	131 mmol/kg	~79–510 μmol/L	[8,17]
	Acetate	44–69 mmol/kg	~5–402 μmol/L	[8,9,17]
	Propionate	14–25 mmol/kg	3.3–ca.15 μmol/L	[9,17,18]
	Butyrate	15–26 mmol/kg	2.1–10 μmol/L	[9,18]
	Valerate	~4.5 mmol/kg	1.3–ca 4 μmol/L	[8,17,18]

**Table 2:** Concentrations used in pivotal studies with in-vitro experiments with Treg/Th17 cells in humans and mice.

Author	Experiment	Cells Used for Assay	SCFA Used	Dose Used	Effect For	No Effect For	Outcome
[48]	Lymphocyte proliferation assay, cytokine production	rat lymphocytes (from lymphnodes)	acetate, butyrate, propionate	acetate 10 mM, butyrate 1.5 mM, propionate 2 mM	Butyrate at 0.25 mM–1.5 mM	acetate, propionate (Il-2)	↓ Thymidine incorporation ↑ IL-10 production after 48 h for acetate, propionate or a combination of both/with butyrate; ↓ IL-10 after 24 h for butyrate alone buyrate inhibited IL-2/IFN- γ production
[22]	Induction of T_reg_ cells in vitro	splenic naïve (CD44^lo^ CD62L^hi^) CD4^+^ T cells	(Sodium) acetate, butyrate propionate	0.1 mM	Butyrate, propionate	acetate	↑ Tregs for butyrate, moderately for propionate, no effect for acetate
[21]	Induction of extrathymic Tregs in vitro	Peripheral naïve (CD44^lo^CD62L^hi^CD25^−^) CD4^+^ T cells	Butyrate, isovalerate, acetate, propionate	0–1024 μM	Butyrate, isovalerate, propionate	acetate	↑ FOXP3+ cells
[6]	Purified cT_regs_ from GF mice cultured for 24 h	cT_regs_ from GF mice	propionate	0.1 mM	propionate	--	↑ IL-10 expression; ↑ IL-10 protein; ↑ FOXP3 expression, no effect for TGF-β
[7]	Effect of SCFAs under; anti-CD3/CD28 in a Th17 or Th1 condition	naive CD4^+^ T cells	acetate, propionate, butyrate	acetate: 0–20 mM propionate: 0–1 mM	Optimal: acetate 5–20 mM; propionate: 0.5–1 mM	acetate (<1 mM), propionate (<0.5 mM)	↑ Th1 and Th17 differentiation ↑ expression of IL-17A, IL-17F, RORα, RORγt, T-bet, and IFN-γ ↑ FOXP3 in low anti-CD3 (1 μg mL^−1^) activation condition ↑ high IL-10 expression ↑ IL-10, IFN-γ, and IL-17 in CD8^+^ T cells
[49]	peripheral blood mononuclear cells (PBMCs)/	Human PBMCS	butyrate, propionate, acetate	Propionate+ acetate: 0–6.4 mM; butyrate: 0–1.6 mM	butyrate, propionate	acetate	↓ expression of CD25 in CD4^+^ and CD8^+^ cells, butyrate (0.1–0.4 mM) ↑ CTLA-4
[50]	Treg & Th17 polarizing conditions	human naive CD4^+^ T cells (CD45RA^−^CD45RO^+^CD25^−^CD127^+)^	propionate	1 μM–10 mM	1 μM–1 mM (Treg), 150 μM Th17	10mM propionate	↑ CD4^+^CD25^+^Foxp3^+^ cells; ↑ proliferation of differentiated Tregs; ↓ CD4^+^IL-17A^+^ T cells
[47]	CD3 stimulation	Human PBMCS	butyrate	0–100 μM,	50 μM and 100 μM	10 μM butyrate	↑ % of Tregs in PBMCs
[51]	CD3 stimulation/Treg polarizing conditions	Murine CD4^+^ cells from lymph nodes/spleen	butyrate, propionate, acetate	0.1–10 mM	Increased regulatory expression: 0.1–0.25 mM; supressed regulation: 0.5 mM–1 mM	acetate, butyrate >0.5 mM induced IFN- γ but not Tregs	≤0.25 mM: ↑ FOXP3 and inducible Treg differentiation only when TGF-ß was suboptimal; absence or presence of high TGF-ß abolished effects of butyrate; >0.25 mM: abolished effect of Treg differentiation, induction of IFN- γ,(not IL-17A or IL-4)

Abbreviations: IL: interleukin; FOXP3: forkhead box P3, ROR: retinoic acid–related orphan nuclear receptor, IFN: Interferon, TGF: transforming growth factor.

**Table 3:** Association studies of SCFA and in humans with autoimmune pathology.

Reference	Groups	SCFA Studied	SCFA Measured In	Concentration in Patient Group	Findings
**Multiple Sclerosis**					
[55]	30 MS/CIS 10 HC	acetate, butyrate, propionate	blood	concentrations in nmol/mL: acetate: ~1600; butyrate: ~14; propionate: ~160	lower propionate in MS than controlsbutyrate positively correlates with Tfh cells no association of SCFAs with Treg, Th1 or Th17 counts
[17]	58 (RR)MS 50 HC	acetate, propionate, butyrate, valerate	blood	concentrations in μmol/L: total SCFA 453; acetate 349; butyrate: ~6.4; propionate ~15 valerate: ~3.8	lower total SCFAs concentration in MS compared to controls no individual difference for acetate butyrate, propionate or valerate between MS and controls after multiple testing correction no significant correlation of SCFAs with inflammatory cytokines in patients with MS positive association of butyrate with TNF and IFN-γ in HC, negative correlation of acetate and IFN-γ SCFA levels not related to clinical follow up at 12 months
[57]	95 MS 54 HC	acetate	blood	approximate mean values in µM/L: acetate: ~25; propionate: ~3.9; butyrate: ~3.8	higher acetate levels in blood of patients with MS; positive association with CD4^+^IL-17^+^ (not significant after correction) and CD8^+^ IL-17* cells, negative association with naive CD4^+^ cells
[58]	34 MS/12 MS for cells 34 HC	propionate, butyrate, acetate	fecal samples	na	depletion of fecal acetate, propionate, and butyrate in MS; fecal SCFA level positively correlated with pTreg frequency
[56]	41 MS/35 controls	propionate, butyrate, acetate	fecal samples	median concentrations in mmol/g: acetate 41.70; propionate 5.51/butyrate 1.25/valerate	non-significantly lower SCFA concentrations in MS compared to controls
**Type 1 Diabetes**					
[59]	19 patients with T1D and matched controls+ animal model	acetate, butyrate	feces	total μg/g: acetate ~1700; butyrate: ~1600	·significantly lower SCFA concentrations (butyrate, propionate acetate) in T1D; valerate levels similar No assessment of T cells in patients; higher frequencies of CD4^+^FN-γ^+^ and CD8^+^TNF-α+ T cells in the mice colonized with fecal bacteria from patients with T1D vs. controls
[60]	132 T1D; 40 HC	propionate, butyrate, acetate	feces	total µmol/g: acetate: 39; propionate: 9, butyrate: 7.8, valerate: 1.8	propionate and butyrate significantly lower in T1D, valerate and acetate not different; higher hsCRP and neutrophil count, correlation with SCFAs not assessed
**Rheumatoid arthritis**					
[61]	82 people with increased risk for RA	acetate, butyrate, propionate or pentanoate	blood	serum in uM: acetate: ~80; butyrate: ~3; pentanoate: ~4.5; Proprionate ~8	higher total sum of SCFAs and/or butyrate/acetate in people who did not develop RA;·butyrate correlated negatively with serum IgA-anti-citrullinated protein antibody levels but not with IgG or IgM isotypes; T cells were not assessed
[62]	19 RA, 20 HC stool/13 HC 14RA blood	acetate, butyrate, propionate	stool and blood	concentrations in stool, μmol/g: acetate: ~7; propionate ~2, butyrate ~1.8; in blood μmol/g: acetate: ~30, proprionate ~2, butyrate ~10	stool: significant reduction in butyrate/propionate, no difference in acetate; serum: no difference in propionate/butyrate but significant increase in acetate; butyrate correlates positively with circulating IL-10+ B cells in serum.
[63]	9HC 10RA	acetate, butyrate, propionate, valerate	stool	concentrations in umol/g: acetate ~9, propionate ~1.5, butyrate ~5; valproate ~0.19	lower acetate, propionate, butyrate and valerate in patients compared to controls
[64,65]	10 HC 36/29 RA	acetate, butyrate, propionate	stool and serum	in mM: in serum: total in mM: ~0.2, acetate: ~0.2, propionate: ~0.015, butyrate ~0.004	no comparison between HC and RA patients regarding SCFAs, ·inflammatory cytokines were not higher at baseline, but patients received DMARD therapy

Abbreviations: Tfh: follicular T helper cells; Th: T helper cell; MS: Multiple sclerosis; RA: rheumatoid arthritis; T1D: Type 1 diabetes; IBD: Inflammatory bowel disease; HC: healthy control; CIS: clinically isolated syndrome; Ig: Immunoglobulin; na: not applicable.

**Table 4:** Interventions with short chain fatty acids in human patients with autoimmune disease assessing changes in T cells.

Reference	Disease	*n* Cases/Controls	Intervention (RCT/Animal/IV)	Intervention+Dose	Duration	Cell Type Studied	Measured SCFA Concentration Post Intervention	Outcome
[37]	MS	91 MS, 24 HC	Short-term propionate	1 g oral propionate daily	14 days	Treg, Th17	in CSF (*n* = 3) ↑ propionate after mean of 2.8 months	↓ Th17, ↑ Treg count, ↑ metabolism and function after 14 days and 90 days; ↑ Tregs suppressive capacity ex vivo; ↓ IL-10 ex vivo cell culture of propionate treated patients, mixed results for IL-17
	MS	52 MS	Long-term propionate	1 g propionate daily/1–3 years	1–3 years	na	na	improved clinical outcome (disability status and relapse rate)
	RA	20 RA	Short-term propionate	1 g propionate daily	14/28 days	Treg	na	↑ Tregs after 14 days
[93]	end stage renal disease	10 ESRD patients/7HC	Open-label intervention study propionate	2 × 500 mg daily sodium propionate	30 days	Treg, CD25^+^CD127-	na	↑ Tregs over time, but not due to proliferation; ↓ after treatment with propionate; no consistent difference for granzyme, IFN-γ, IL-2, IL-17, and TNF-α producing CD4^+^ and CD8^+^T-cells
[35], sample from [55]	MS	34 non obese MS (22 at fu); 6 obese MS (5 at fu)	propionate	500 mg sodium-propionate (PA) capsules twice daily	90 days	Treg, Th17	na	↑ Tregs in non-obese group, non-significant ↑ in obese group; ↓ Th17 in obese group, non-significant ↓ in non-obese group
[70]	T1D	20 T1D	HAMSAB dietary supplement	40 g/day HAMSAB	6 weeks	T cells, B cells, monocytes	plasma: ↑ acetate after 3 weeks, maximum at 6 weeks: 58% subjects acetate ↑ >2-fold; similar but less strong observations for propionate + butyrate;	↑ (naive)B cells at week 6; ↓ CD86 on B cells after washout period of 6 weeks; ↑ CD3+ cells after washout period week 12; ↑ CTL4A expression; ↓ plasma IL-8, MIP-1α, and bFGF at week 12 compared to baseline
[71]	T1D	30 T1D	RCT with butyrate/placebo	4 g sodium butyrate or placebo	1 month	PBMCs (monocytes, autoreactive CD8^+^ T cells)	← fecal acetate, ↓ fecal propionate and butyrate	← CRP after butyrate treatment; ← monocyte subsets (compared to placebo) ← ex vivo cytokine production; ↓ islet autoreactive CD8 T cells (assay only possible in in 13/30); ← lymphocyte subsets
[64,65]	RA	36 RA	dietary intervention high fiber bars	high-fiber bars 15–30 g/day or cereals	28 days (14 + 14)/30 days	Tregs, Th1, Th17	↑ acetate and butyrate and propionate after 30 days	↑ Tregs, ↑ Th1/Th17 ratio; ← CD4 and CD8 numbers. Improved physical & mental quality of life and disability scores, ↓ IgA; ← anti-citrullinated vimentin p18 peptide antibody levels; ← for IL-1β, IFN-a2, IFN-γ, TNF-α, IL-6, CXCL-8 (IL-8), IL-10, IL-12p70, IL-17A and IL-23; ↓ for CCL-2, IL-18 and IL-33

Abbreviations: ↑ increase in; ← no change in; ↓ IgA: decrease in; CSF: cerebrospinal fluid; HAMS/A/B/P: high-amylose maize-resistant starch modified with acetate/butyrate or propionate, CTL4A: cytotoxic T-lymphocyte-associated Protein 4, MIP: Macrophage Inflammatory Proteins, and bFGF: basic fibroblast growth factor, RCT: randomized controlled trial; PBMC: peripheral blood mononuclear cells; CRP: C-reactive protein; CXCL: chemokine (C-X-C motif) ligand; CCL: chemokine ligand, na: not applicable.

## Data Availability

All Data is reported in the manuscript/no analysis was performed on original data.
